# Plague and Climate: Scales Matter

**DOI:** 10.1371/journal.ppat.1002160

**Published:** 2011-09-15

**Authors:** Tamara Ben Ari, Simon Neerinckx, Kenneth L. Gage, Katharina Kreppel, Anne Laudisoit, Herwig Leirs, Nils Chr. Stenseth

**Affiliations:** 1 Centre for Ecological and Evolutionary Synthesis (CEES), University of Oslo, Oslo, Norway; 2 Ecole Normale Supérieure, CNRS UMR 7625, Paris, France; 3 Bacterial Diseases Branch, Division of Vector-Borne Diseases, Center of Control and Prevention, Fort Collins, Colorado, United States of America; 4 Liverpool University Climate and Infectious Diseases of Animals Group (LUCINDA), Department of Veterinary Clinical Sciences, University of Liverpool, Leahurst, Great Britain; 5 Evolutionary Ecology Group, Department of Biology, Universiteit Antwerpen, Antwerp, Belgium; University of California San Diego, United States of America

## Abstract

Plague is enzootic in wildlife populations of small mammals in central and eastern Asia, Africa, South and North America, and has been recognized recently as a reemerging threat to humans. Its causative agent *Yersinia pestis* relies on wild rodent hosts and flea vectors for its maintenance in nature. Climate influences all three components (i.e., bacteria, vectors, and hosts) of the plague system and is a likely factor to explain some of plague's variability from small and regional to large scales. Here, we review effects of climate variables on plague hosts and vectors from individual or population scales to studies on the whole plague system at a large scale. Upscaled versions of small-scale processes are often invoked to explain plague variability in time and space at larger scales, presumably because similar scale-independent mechanisms underlie these relationships. This linearity assumption is discussed in the light of recent research that suggests some of its limitations.

## Introduction

**Figure 1:**
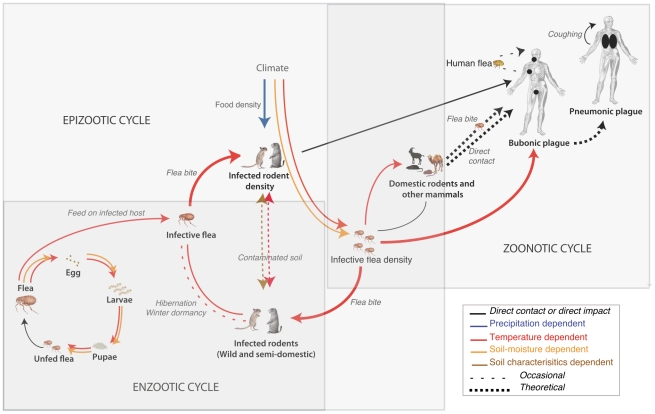
Schematic of the plague cycle with small mammals as hosts and fleas as vectors. Arrows represent connections affected by climate with a color-coding depending on the most influential climate variable on this link (i.e., precipitation, temperatures, and other variables indirectly depending on them such as soil characteristics and soil moisture). Grey rectangles somewhat arbitrarily delimit epizootic, enzootic, and zoonotic cycles. Note that despite their location at the far end of the cycle, humans often provide the only available information on plague dynamics.

Plague is a rapidly progressing infectious disease that is infamous for having caused the death of millions of people in large historic pandemics [1] as well as numerous other deadly but localized outbreaks [2]. Plague, caused by the pathogenic bacterium *Y. pestis* is transmitted from host to host by fleas via blood feeding, through consumption or handling of infectious host tissues, or through inhalation of infectious materials. Plague is thought to persist for long periods of time at low to very low levels of prevalence in so-called enzootic cycles that cause little host mortality and involve partially resistant rodents (often called enzootic or maintenance hosts). These long periods are punctuated by occasional outbursts or epizootics (i.e., spreading die-offs) among these hosts or epidemics, when the incidence among humans increases. Figure 1 illustrates these intertwined cycles.

Climate has long been suspected to be a key factor in the alternation between quiescent and active periods of plague. Rogers (1928) suggested seasonal variations in temperature and humidity to be responsible for the seasonal patterns of human plague incidence in India [3]. Decades later, Davis showed that human plague outbreaks in several African countries were less frequent when the weather was too hot (>27°C) or cold (<15°C) [4]. Subsequent studies showed an increased plague incidence in Vietnam during the hot, dry season, when following a period of high seasonal rainfall [5], [6]. Nowadays, several studies, as we report here, demonstrate climate's impacts on plague incidence spatial and temporal variability.

In the context of public health and wildlife conservation, we need an improved understanding of the mechanisms underlying the association between plague outbreaks and climate. As we will show in this review, this understanding is only partially available at present. There are several reasons for this. First and perhaps most importantly, the plague cycle is complex. It is composed of three components that interact with each other and all are influenced by climate variables with a broad range of times lags. Also, climate variability manifests itself at numerous temporal and spatial scales that condition the form of the response in plague dynamics. To cope with this series of difficulties, we break down the problem as follows: in section 1 we review individual effects of climate variables on each of the plague components. Our knowledge of these effects is primarily based on small-scale studies that are useful because they provide conceptual models for how larger scale climate variability may force the plague system. The way these conceptual models are most often used raises the issue of upscaling conclusions by inference from the results of small scale studies, a subject on which we focus on in section 2. Also, in the latter we review likely impacts of climate change on plague incidence.

## Climate Dependence of the Flea Vectors and Rodent Hosts

The plague system is the result of complex interactions between its components, the densities, life cycle, dynamics and geographical distributions all of which are individually influenced by climate variables. Climate variables influence the dynamics of flea vectors and rodent hosts with responses varying considerably among species [7], [8]. Figure 1 illustrates the plague cycle in relation to those climate variables known to be important (namely temperature, humidity and precipitation) to the main plague hosts and vectors.

It is accepted that abundance of rodent fleas is affected by ambient temperature, rainfall, and relative humidity, with warm-moist weather providing a likely explanation for higher flea indices [4], [5]. Indeed, temperature, rainfall, and relative humidity have direct effects on development and survival, as well as the behavior and reproduction of fleas and their populations [9]–[12]. For example, the rate of metamorphosis of *Xenopsylla cheopis* (as a primary flea of the black rat *Rattus rattus*, *X. cheopis* is likely the main vector of plague in foci affecting humans), from egg to adult is regulated by temperature. Fleas are ecto-thermic and hence sensitive to temperature fluctuations; this is enhanced by the fact that all of the immature flea stages occur off host. Flea development rates increase with temperature until they reach a critical value; then the survival of immature stages decreases if high temperatures are combined with low humidity [13]. Temperature and relative humidity impact flea survival [5]: survival is in fact inversely proportional to air saturation deficit at a constant temperature [14]. Flea larvae are susceptible to desiccation [15] and typically acquire water from adult excreta. Survival of immature stages of fleas in rodent burrows is also affected by soil moisture that is partly controlled by outside precipitation [16] even though detrimental moisture losses and temperature swings are reduced by living underground [9]. Conversely, when coupled with a high organic load, excessively wet conditions in rodent burrows (*e.g.,* relative humidity >95%) can promote the growth of destructive fungi that diminish larval and egg survival [5], [17].

Rodent survival and population dynamics are also affected by climate. A direct effect occurs when high intensity rainfall causes flooding of rodent burrows [5] but the effects of precipitation on rodent densities are mostly bottom-up [18]. Indeed, rainfall controls primary production which limits rodent abundances [19]. Reproduction and recruitment periods often follow wet seasons when increases of primary production can be used to build up juvenile populations [20]. Accordingly, rodent population densities show clear association with annual rainfall and its seasonal distribution e.g., in Chile [21]–[23], Tanzania [24], and Australia [25], [26]. But the relation between precipitation patterns and rodent densities can be complex, localized, and dependent on the timing and the intensity of precipitation events (see also below) [8], [27]. Temperature effects on rodent populations are less clear in part because rodents are homeothermic and hence do not respond immediately to changes in ambient temperatures. In temperate areas, low temperatures in winter can nonetheless negatively affect rodent populations either directly or through low food availability [28]. Nevertheless, under some circumstances, conditions detrimental to hosts or vectors can favor the maintenance of plague. Evidence of hibernation as a factor of prolongation or modification of *Y. pestis* infection in rodents would need further elucidation. The flea *Citellophilus tesquorum altaicus* for instance, is supposedly able to maintain a plague infection over the winter by feeding on hibernating long-tailed Siberian souslik (*Citellus undulatus*) [29]. Also, populations of *Tatera indica* aestivating during adverse conditions in India presumably continue to act as hosts for infected fleas, thereby promoting the persistence of plague infection within the area [30]. Hence, the survival of *Y. pestis* in relatively plague-resistant burrowing rodents that interrupt their activities to hibernate through winter or aestivate in summer could influence or prevent the transient temporal and spatial extinction of plague occurrence.

### Human Plague Incidence Is Not Unrelated to Human Factors

Human activities and behavior in plague-infected areas are also to be considered as important determinants of plague transmission to humans [8]. When occurrences of plague are due to human intrusions in natural plague areas, it is thus important to consider climate as a second order variable that influences disease incidence through human behavior (drought, famine, war, or other events). In Argentina, plague transmission reportedly occurred during the harvesting season [31]. In Lushoto, a plague endemic region in Tanzania, daily and gender activities seem to impact plague levels [32], although plague tends to peak during the season with the least agricultural activity, which is a time when people usually gather in houses.

### What Plague Niches Reveal about Plague's Environmental Preferences

Plague foci are present over an expanded geographical range that includes the Western US, portions of South America, East and South Africa, and Southeast Asia [33]. Long-term maintenance of plague in defined ecological niches may inform us about the environmental conditions that are required for plague to establish in permanent foci. Unfortunately, enzootic plague is poorly described; in many foci, local reservoirs have yet to be identified. The geographical limits of plague territories are hence rarely defined or only by occurrence in domestic rodents and humans [34]. It is reasonable to assert that plague manifests itself under various ecological conditions [35], [36]. It is noteworthy though, that modern plague foci in North and East Africa, Western North America, parts of South America, and many scattered regions in Asia (notably China and Kazakhstan) occur primarily in either semi-arid to arid areas or low humidity forest types of habitat, and the disease apparently fails to persist for long periods in humid tropical lowland areas (except from occasional invasion through movement of infected humans or transportation of infected rodents or fleas) [37]–[39]. Also, plague is almost invariably absent from the hottest and driest desert regions like the Sahara or Sonoran deserts [37], [40].

## The Complexity Introduced by Interactions among Scales and Other Nonlinearity

**Figure 2:**
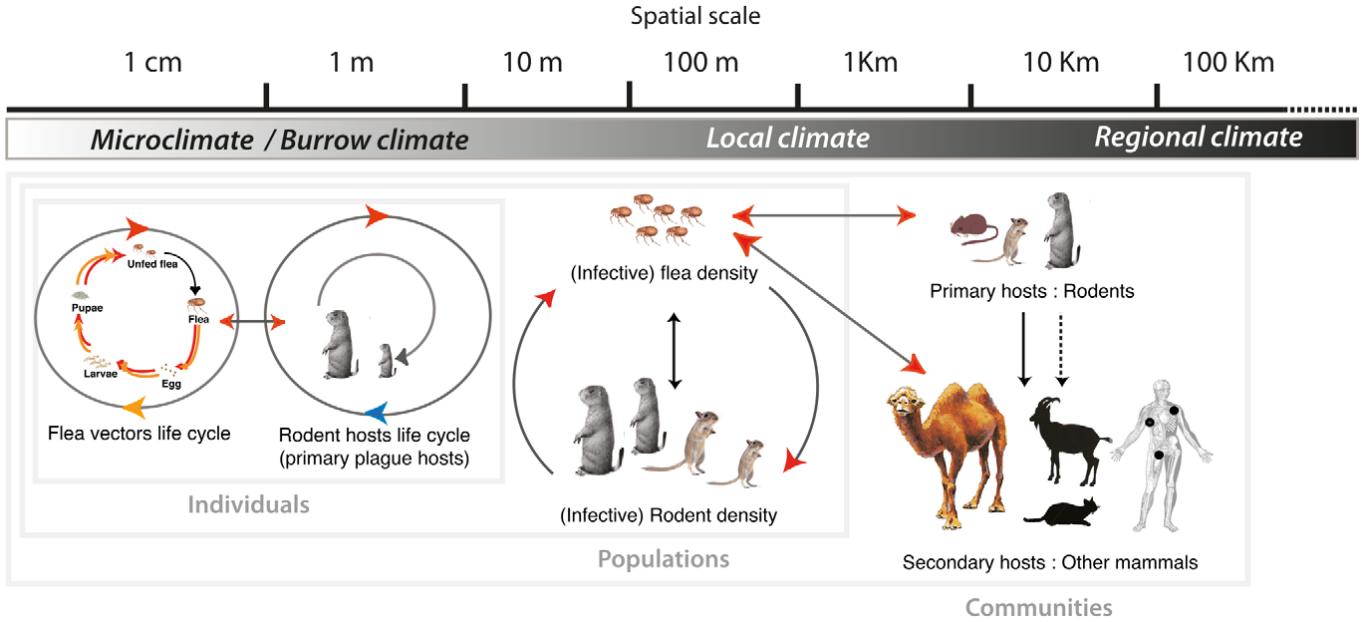
Illustration of the abiotic environment impact on the plague cycle as a function of spatial scale. Arrows represent connections affected by climate (see Figure 1 for the meaning of color coding). Most climate variables act over a wide range of scales and only the effects we deemed most important are represented. At the level of individuals, populations and communities hosts and vectors are influenced by climate variability at the relevant scale (local or regional). At the smaller scale, the burrow acts as a filter on climate variables. Note that secondary hosts are placed at the kilometer and larger scale on the basis of the type of information generally available regarding their infection.

The previous section reviewed reported effects of climate variables on the components of the plague cycle, indicating that climate affects rodents and fleas individually and their population dynamics and structures. Consequently, climate impacts the natural cycle of plague as a whole and in ways that are not likely to reflect simply the sum of these individual effects. In this section, we review studies on the effects of climate (including the environment) on plague at different spatial levels and occurrences ranging from rodent burrows to areas that form a coherent and apparently self-sustaining system (a single niche or focus), or even to larger regions that could comprise many such systems (several foci). We emphasize the fact that (i) scales relevant to plague ecology are nested within each other, as shown in Figure 2, so that climate effects at a given scale may not be simply extrapolated from those observed at a smaller scale, and (ii) processes exist that prevent the plague system from responding to climate fluctuations in a way that simply reflects the sum of the responses of its individual component.

It is readily apparent that associations exist between large-scale climate such as climate indices and plague/plague hosts dynamics; associations that can be consistently explained by processes detailed in the first section. Effects of precipitation patterns on plague incidence are for instance assumed to be the results of climate's bottom-up effects on plague hosts. In Peru, climate fluctuations associated with El Niño were related to a bubonic plague outbreak in 1999 [41]. In northern Colorado, prairie dog colony extinctions attributed to plague were weakly associated with El Niño southern oscillation [42]. In the Western US, spatial synchrony of periods with low and high number of human plague occurrences throughout the west revealed large scale synchrony [43]. In most foci of the southwestern part of this region, above normal precipitation in winter and spring was used to explain increases in human plague cases [44], and high summer temperatures, decreases of its incidence in the same area [45]. The El Niño/Southern Oscillation (ENSO) and the Pacific Decadal Oscillation were similarly related to precipitation, temperatures, food availability, and the number of plague cases themselves [46]. Finally, the increase rate of human plague in China is associated at the province level with the Southern Oscillation Index and Sea Surface Temperature of the tropic Pacific east equator [47]. An important assumption to the above-cited studies is that large-scale climate variability produces coherent and synchronous effects on rodent hosts populations. Few studies actually investigate the reality of the invoked synchronous trophic forcing on plague hosts' population dynamics so as to demonstrate such a climate induced bottom-up control. At this point, it is worth mentioning a counterexample provided by Kausrud et al. [48], who investigated the population dynamics of great gerbils *Rhombomys opimus*—the main plague host in Kazakhstan—and demonstrated spatial synchrony of their populations over areas larger than the ones expected by migration processes. Their results indicate that the observed synchrony of great gerbil's densities was most probably due to the effects of climate and that similar bottom-up processes could also synchronize plague activity in this focus.

In any case, there are caveats to inferring processes occurring at small scale as a means to explain the ones observed at larger scales. First, causal relations can typically only be suggested (e.g., by a chain of supposedly causal processes from climate indices to relevant climate variables and from these to plague incidence) but not demonstrated. More problematically, a variety of processes can be expected to interfere with each other in the transition from small to large scale. Tentatively, we propose below a classification of these processes into two categories. The first ones pertain to the complexity of the environment itself and the second ones to population interactions.

An example in the first category can be provided by studies showing that rodent burrows could be considered a “climate filter”. Conditions in rodent burrows are subject to environmental factors from nearby surroundings such as temperature, humidity, vegetation, various soil properties (e.g., soil texture and structure, soil organic carbon content, etc.), and other landscape factors (e.g., slope, orientation with respect to dominant winds, sun exposure, etc.). But, the underground location of most burrows implies that conditions inside these structures fluctuate only moderately [49], [50]. In fact, temperatures inside and outside burrow systems are highly correlated, but inside humidity is a complex function of past rainfall and soil characteristics [51] rather than of present ambient humidity [52]. Note that investigations on burrow micro-climate have been conducted in different parts of the world [51], [53], [54], including in plague-endemic regions, but these measurements were generally obtained from only a small number of natural or, in some instances, artificial burrows, allowing investigators to draw only tentative conclusions from the results of these studies [49], [55]. Perhaps more importantly for our present purposes, climate conditions inside the burrows could specifically influence the distribution of plague vectors so that hosts' distribution becomes insufficient to explain vectors distribution, a situation that has been observed in plague areas in Madagascar [56]–[58] or plague-free areas in Israel [59], [60]. These examples emphasize the difficulty of upscaling processes by simple extrapolation. Among the numerous other examples of environment-related complications are the landscape heterogeneities within plague areas. In particular, strong orographic forcings frequent in plague areas locally affect large-scale climate variability [61]. In fact, accurate plague prediction models seem to require high resolution environmental and climate representation (e.g., 250-m resolution images accurately predict plague distribution when 10-km resolution images fails in sub-Saharan Africa). According to the authors, plague focality can not be explained by fragmented environmental conditions at this coarse scale [35], [62].

In the second category are the complications introduced by interactions within and between plague host and plague vector populations in their response to climate variability. It is commonly expected that fluctuations in abundance of rodent hosts, e.g. induced by climate variability, will translate into plague prevalence fluctuations. However, the relationship between host abundance and host prevalence is complex and scale dependent. Field studies of rodent reservoirs show a negative correlation between host abundance and host prevalence at seasonal time scale. The finding has been explained by the juvenile dilution effect, that is, the arrival of numerous healthy juveniles in a population [63]; an effect likely to be relevant for diseases with no vertical transmission such as plague (or Hantavirus [64]). In contrast, a few longer field studies (>5 years) show a positive, although delayed relationship between reservoir abundance and prevalence [63]. These antagonist responses to variability at different time scales are typical of systems in which nonlinear interactions play an essential role. The different responses of rodent and flea populations to climate (fast for fleas while rodents tend to integrate environmental conditions over some years) provide another reason for questioning the existence of a straightforward link between abundance and prevalence. A more complex model was proposed for the Western US, in which human plague incidence depends both on time-lagged precipitation events, which presumably increase rodent numbers and favor plague epizootic, and on relatively cool summer temperatures during the plague transmission season, which are favorable to infectious fleas [45]. This model has been coined “trophic cascade hypothesis,” although it has yet to be tested rigorously for plague under field conditions [65] (see also [66] for a discussion on the accuracy of the use of the term “trophic” for the cascade hypothesis). In particular, the scale at which the trophic cascade model is valid needs to be addressed. Parmenter [44], who first introduced this model, shows its relevance at a local scale (i.e., by demonstrating significant associations between plague and nearby precipitation), but could not extend this result to a state-wide level. Interestingly, a cascade relationship was recovered at an even larger scale by Ben-Ari [46], possibly because the integration of delayed density-dependence effects of large-scale climate on rodents were taken into account at decadal time scales. The study by Stenseth et al. [67] illustrates the challenge that needs to be confronted when addressing cascading effects of climate on plague prevalence in nature. There are numerous relationships between climate elements (temperature and precipitation with or without lags) and various ways these elements can impact the plague components (in this particular case rodent density, prevalence, and flea burden). The type of dataset that would let us isolate/untangle the mechanisms and the spatio-temporal scales at which processes operate may not be available.

These examples do not necessarily invalidate studies that scale up small-scale results (individual measurements, lab experiments, local correlations, etc.) in the simplest way, but we believe they illustrate the need for more investigation on the impact of complex interactions and environment heterogeneities at intermediate scales.

## Implications for Climate Change

The need to understand disease responses in relation to climate variability is made particularly acute by ongoing global climate change. Effects of temperature rise on vector-borne diseases and notably the ones involving free-living stages of terrestrial animals are expected [68]. Beyond that, a lot of uncertainty remains on whether or how climate change might affect pathogens and disease transmission, i.e., changes in population size (for vectors or hosts), overall prevalence, timing and seasonality, or shifts in geographical distribution. There are various choices to be made when addressing climate change impact on a disease like plague, particularly with respect to the degree of complexity that should be chosen for climate models and any associated biological models and the relation between them.

Stenseth et al. [67] performed a sensitivity study to a one-degree increase in temperature into a statistical host vector plague model developed specifically for Kazakhstan. They show that this (simple) climate change scenario would lead to a 50% increase in plague prevalence among great gerbils. Nakazawa et al. [69] used two Global Circulation Models (GCMs) to infer mean temperature and precipitation changes between the present and a 30-year period centered around 2055. These changes were then applied to a higher resolution present state GCM that provided a climate changed forcing state, which was fed into an Ecological Niche Model (ENM) predicting plague occurrences in the US from a set of environmental variables. They show subtle shifts of plague habitats (generally northward). Snall et al. [70] used an elaborate procedure to downscale climate scenarios from several GCMs before using these data to force an explicit model for the joint host-parasite dynamics of black-tailed prairie dogs and plague in the Western US. A related decrease in the number of infected prairie dog colonies (leading to an increase in prairie dog colonies) is predicted, presumably, as a consequence of the negative impact of increased frequency of abnormally hot days on plague transmission.

These studies are difficult to compare with each other because of the specificities of their methodologies even when they lead to contrasting results over the same region [70]. Admittedly, more investigations are required. Among the numerous possible approaches, the safest arguably would rely on using climate-forcing sensitivities of intermediate complexity where biological models are forced by existing modes of climate variability. This makes sense not only because climate change will in large part occur through a modification of these modes (which can be extracted from Intergovernmental Panel on Climate Change [IPCC] GCMs [61]), but also because biological data are then available for evaluation.

## Conclusion

Climate impacts all components of the plague cycle (host, vector, and pathogen) in various ways and over a wide range of scales (from micro—individual flea life cycle—to macro—a plague area composed of several disjoint foci). Several studies have established links between plague occurrence and climate factors that can a posteriori be justified by assuming the validity at a large scale of relationships that have only been observed at a small scale (assumption of linearity). We have reviewed both the successes and the limitations of this assumption. To go beyond simple inferences on how climate fluctuations (including long-term climate change) affect plague, it might be necessary to select a (or a few) preferential scale, on the basis of the fact that they would be the most informative and/or relevant for public health policies. In this regard, intrinsic dynamics of plague hosts and vectors should be kept in mind as it alone greatly contributes to the entanglement of scales that drive the overall dynamics of plague prevalence [71]. Further, assessing plague risks for humans at such scales may in fact require investigating plague dynamics on a much wider range of scales and presumably include a fuller description of the plague system in its environment, as we have tried to outline in this review.
