# Correction: Plague and Climate: Scales Matter

**DOI:** 10.1371/annotation/84f83f75-2e53-48cf-9f43-7e5eaed74437

**Published:** 2012-05-07

**Authors:** Tamara Ben-Ari, Simon Neerinckx, Kenneth L. Gage, Katharina Kreppel, Anne Laudisoit, Herwig Leirs, Nils Chr. Stenseth

The first author's name was spelled incorrectly. The correct name is: Tamara Ben-Ari. The correct citation is: Ben-Ari T, Neerinckx S, Gage KL, Kreppel K, Laudisoit A, et al. (2011) Plague and Climate: Scales Matter. PLoS Pathog 7(9): e1002160. doi:10.1371/journal.ppat.1002160

